# Veterinary support staff knowledge and perceptions of antimicrobial drug use, resistance, and stewardship in the United States

**DOI:** 10.3389/fvets.2024.1401290

**Published:** 2024-08-07

**Authors:** Lauren Gunn-Sandell, Daniel D. Taylor, Elaine Scallan Walter

**Affiliations:** Colorado Integrated Food Safety Center of Excellence, Colorado School of Public Health, Aurora, CO, United States

**Keywords:** antibiotic resistance, antibiotic use, stewardship, veterinary staff, companion animals

## Abstract

Antimicrobial drug use (AMU) in veterinary medicine may contribute to antimicrobial resistant (AMR) infections in both animals and people. Efforts to improve AMU in companion animal medicine are underway and should include all members of the veterinary team, including veterinary support staff. Our objective was to describe knowledge and attitudes regarding AMU, AMR, and antimicrobial stewardship (AMS) in companion animal medicine among veterinary support staff professionals in the United States using an anonymous, online questionnaire. Additionally, we sought to explore veterinary support staff perceptions of their role in the antimicrobial drug (AMD) prescribing process. Veterinary technicians, nurses, assistants, client care representatives, and hospital managers (*n* = 337) considered AMR a global concern (83.4%), and 40% reported receiving AMR education from their employer. Few (18.3%) were aware of AMS, with only 6.4% indicating that their clinic had an AMS program. Frequent involvement in the AMD prescribing process was reported (43.4%), but only 19.7% perceived involvement with AMS interventions. Approximately one-third of participants (34.9%) said that advice regarding the need for AMDs was routinely provided by staff to pet owners prior to veterinary consultation. Participants estimated that 82.6% of all AMD prescriptions were filled at the clinic as opposed to an outside pharmacy. Given their direct involvement in the AMD prescribing process and frequent interactions with pet owners, AMS should be emphasized to all veterinary staff. Involving support staff in AMS interventions is necessary to improve AMU in companion animal medicine.

## Introduction

Globally, antimicrobial resistance (AMR) is a critical animal and public health problem. In the United States alone, antimicrobial resistant pathogens are estimated to complicate three million cases of human illness annually, resulting in 35,000 deaths (1). In companion animal medicine, AMR impacts animal health and welfare due to infections that do not respond to antimicrobial drugs (AMD) (2, 3). The development of AMR has been linked with antimicrobial drug use (AMU) in both people and animals (4). Moreover, a previous human drug-resistant enteric disease outbreak traced back to puppies has raised questions about how AMU in companion animals contributes to AMR in humans (5, 6). As such, mitigation strategies to reduce AMR in humans and animals require a One Health approach integrated across human, animal, and environmental health sectors (7).

Numerous efforts are underway to promote judicious AMD use principles in veterinary medicine (8, 9). Studies focused on companion animal medicine and AMD prescribing suggest that compliance with judicious AMU principles could be improved through the implementation of antimicrobial stewardship (AMS) programs at the clinic level (10, 11). Furthermore, the results from a nationwide survey indicate that veterinarians think support staff training on AMR and AMS can improve AMU in companion animal medicine (12). Veterinary technicians/nurses, assistants, client care representatives, hospital managers and other support staff play a vital role in the everyday operations of a veterinary clinic. Given the team approach to daily clinical operations and their frequent interactions with pet owners, successful AMS interventions need to involve all staff members.

The decision to prescribe an AMD is a complex medical and social process that includes multiple stakeholders, including veterinarians, pets, pet owners, and veterinary support staff. To better understand the dynamics of AMD prescribing and to identify opportunities for improved AMU, all stakeholders need to be considered. However, knowledge of AMU, AMR, and AMS among veterinary support staff, along with perceptions of their role within the AMD prescribing process, has not been assessed. The purpose of this study was (1) to explore veterinary support staff knowledge and attitudes regarding AMU, AMR, and AMS in companion animal medicine, and (2) to assess veterinary support staff perceptions of their role in the AMD prescribing process.

## Materials and methods

We surveyed veterinary support staff who worked in companion animal practice in the United States, using an anonymous, open, online survey designed using Qualtrics™ software. For this study, veterinary support staff included technicians, nurses, other clinic staff (excluding veterinarians), veterinary technician or assistant students, groomers, client care representatives (i.e., front desk staff), and hospital managers. The survey included questions on participant demographics (e.g., veterinary support staff role, years in practice, and location of practice), knowledge and attitudes of AMU, AMR and AMS programs, and perceptions of involvement in the AMD prescription process (Supplementary material S1). Ten questions used a five-point Likert scale, with response options ranging from strongly disagree, disagree, neither agree nor disagree, agree, strongly agree. “Do not know” and “Not applicable” response options were also included. Four additional questions used a four-point scale with options “Yes”, “No”, “Unsure”, and “Prefer not to answer”. Finally, using a sliding scale from zero to 100 %, the last question asked participants to quantify the percent of AMD prescriptions that were filled outside of the respondent’s hospital.

The survey was piloted with 10 veterinary science professionals (i.e., veterinarians, veterinary technicians, and veterinary assistants) with feedback being used to revise the tool prior to its nationwide distribution. The survey was available from February 1, 2021, to April 30, 2021, and a convenience sampling approach was used to maximize the number of responses from veterinary support staff professionals across the United States. State veterinary technician associations in 41 states were contacted via email or social media and asked to distribute the survey link to their members by email, electronic newsletter, or social media post. In the nine states without a veterinary technician association, we contacted the state’s veterinary medicine association for assistance in distributing the survey. Additionally, national associations for veterinary technicians and canine groomers, veterinary social media influencers, and educational publications were engaged to disseminate the survey link. Support staff were eligible to participate if they worked in a veterinary setting or were currently in a veterinary training program based in the United States. Participants self-selected into the study by opening the common distributed survey link and acknowledged informed consent to participate prior to completing the survey. No personal or identifying information was collected. The study protocol and survey instrument were reviewed by the Colorado Multiple Institutional Review Board (COMIRB) and designated as “Not Human Subject Research”. All methods and procedures were administered in compliance with applicable guidelines and regulations.

Demographic characteristics of participants were analyzed descriptively. For analysis purposes, participants were classified into three categories according to their response to the question about veterinary support staff position type: (1) veterinary technician or nurse, (2) veterinary technician student or intern working in a clinic environment, and (3) other support staff, such as hospital administrators or veterinary assistants. When the response to the role question was complete, surveys with partial responses to the perceptions and knowledge items were retained in the final dataset to maximize the sample size for each question. Differences in responses to Likert scale and yes/no questions by role type were examined using a Pearson χ^2^ test (or Fisher’s Exact test when less than five responses) with a *p*-value of <0.05 considered statistically significant. All analyses were performed after the survey period closed using SAS^®^ software version 9.4 (SAS Institute, Cary, NC, United States).

## Results

A total of 367 surveys were returned over the study period. Of these, 30 were excluded because the participant did not work in the veterinary field (*n* = 11), did not report a role (*n* = 5), reported an ineligible role (e.g., veterinarian) (*n* = 8), did not work in the United States (*n* = 4), or did not consent to complete the survey (*n* = 2). Of the 337 participants included in the study, most (60%) self-described as a veterinary technician or nurse, followed by technician student or intern (23%) (Table 1). Most worked in an urban (27%; 90/344) or suburban (47%; 156/344) setting. All four U.S. census regions were represented, although most participants were from the South (38%; 122/321) or West (29%; 92/321). Over half (54%; 178/334) of the participants had less than 5 years of experience. Of those who reported working as a “veterinary technician,” “veterinary nurse,” or “hospital management,” 84% (202/241) had received formal training for their position, such as completing a veterinary technician or veterinary assistant program.

**Table 1:** Descriptive characteristics of study participants in a convenience sample of small animal clinics/hospitals support staff in the US using an online survey tool, 2021 (*n* = 337).

Characteristic	*n* (%, standard error (SE))
*Role (n = 337)*	
Technician or nurse	203 (60, 2.67)
Student or intern	77 (23, 2.29)
Technician assistant	39 (12, 1.77)
Other support	18 (5, 1.18)
*Location type (n = 334)*	
Urban	90 (27, 2.43)
Suburban	156 (47, 2.73)
Rural	63 (19. 2.15)
Unsure	22 (7, 1.40)
Prefer not to answer	3 (1, 0.54)
*Census region (n = 321)*	
Northeast	50 (16, 2.04)
Midwest	57 (18, 2.14)
South	122 (38, 2.71)
West	92 (29, 2.53)
*Years of experience (n = 334)*	
< 1	42 (13, 1.84)
1–5	136 (41, 2.69)
6–10	53 (16, 2.01)
11–15	28 (8, 1.48)
> 15	75 (22, 2.26)
*Any formal training (n = 241)* ^a^	202 (84, 2.36)

a Formal training such as completing a veterinary technician program, veterinary assistant program, or other.

Most participants agreed that they were familiar with AMR (86.5%; 270/312) and that AMR is a global concern (83.4%; 257/308); however, only 32.8% (102/311) indicated that they were concerned about AMR at their clinic or hospital (Table 2). While agreement did not differ significantly between roles, students and interns working in a clinic environment were less likely to agree with these statements when compared with technicians/nurses and other support staff.

**Table 2:** Veterinary support staff knowledge and perceptions of antimicrobial use, resistance, and stewardship by role in a convenience sample of US small animal clinics/hospitals using an online survey tool (2021).

	Agree/strongly agree or yes, *n* (%, SE)				*p*-value
	Technician/nurse^b^	Student/intern^b^	Other support^b^	Overall^b^	
**Antimicrobial resistance statements**					
I am familiar with antibiotic resistance (*n* = 312)	172 (88.7, 2.27)	54 (78.3, 4.96)	44 (89.8, 4.32)	270 (86.5, 1.93)	0.072
AMR is a global concern (*n* = 308)	160 (84.2, 2.64)	54 (78.3, 4.96)	43 (87.8, 4.67)	257 (83.4, 2.12)	0.353
I am concerned about AMR at my clinic/hospital (*n* = 311)	65 (33.7, 3.40)	18 (26.1, 5.28)	19 (38.8, 6.96)	102 (32.8, 2.66)	0.321
I have received education focused on antibiotic use (i.e., from your employer) (*n* = 298)^a^	83 (44.4, 3.63)	23 (35.9, 5.99)	19 (40.4, 7.15)	125 (42.0, 2.86)	0.496
**Antimicrobial stewardship awareness statements**					
I am familiar with antibiotic stewardship programs (*n* = 311)	44 (22.8, 3.01)	8 (11.6, 3.85)	5 (10.2, 4.32)	57 (18.3, 2.19)	0.033
My clinic/hospital has an antibiotic stewardship program (*n* = 298)^a^	13 (7.0, 1.87)	5 (7.8, 3.35)	1 (2.1, 3.53)	19 (6.4, 1.42)	0.433
**Antimicrobial drug prescribing statements**					
I am confident educating clients about antibiotic use in their pets (*n* = 311)	165 (85.5, 2.53)	38 (55.1, 5.98)	37 (75.5, 6.14)	240 (77.2, 2.38)	<0.0001
I am comfortable collaborating with veterinarians and other staff regarding antibiotic use (*n* = 301)	118 (62.8, 3.52)	38 (58.5, 6.11)	30 (62.5, 6.98)	186 (61.8, 2.80)	0.822
I have a role in antibiotic stewardship interventions at my clinic/hospital (*n* = 300)	45 (24.1, 3.12)	6 (9.2, 3.58)	8 (16.7, 5.38)	59 (19.7, 2.30)	0.030
Veterinarians listen to my input when prescribing antibiotics (*n* = 301)	67 (35.6, 3.49)	10 (15.4, 4.47)	14 (29.2, 6.56)	91 (30.2, 2.64)	0.009
I have an impact on whether an antibiotic is prescribed to an animal during a veterinary visit (*n* = 298)	50 (27.0, 3.26)	14 (21.5, 5.09)	14 (29.2, 6.56)	78 (26.2, 2.54)	0.602
I am involved in the antibiotic prescription process at my clinic/hospital (*n* = 298)^a^	95 (50.8, 3.65)	16 (25.0, 5.41)	18 (38.3, 7.09)	129 (43.3, 2.87)	0.001
Advice about whether a pet needs antibiotics is routinely given to a client at the time of making an appointment at my clinic/hospital (*n* = 298)^a^	57 (30.5, 3.36)	26 (40.6, 6.13)	21 (44.7, 7.25)	104 (34.9, 2.76)	0.106

a Question response framework was Yes/No/Unsure/Prefer Not to Answer, ‘yes’ answers are included in table.

b Number of responses varied for each question by role and overall.

Approximately 40% (125/298) of participants reported receiving AMD-specific education from their employer (technician/nurse 44.4%, student/intern 35.9%, other support staff 40.4%, *p* = 0.008). However, only 18.3% (57/311) strongly agreed or agreed that they are familiar with AMS programs (technician/nurse 22.8%, student/intern 11.6%, and other support staff 10.2%, *p*-value = 0.03). Additionally, few (6.4%; 19/298) participants indicated their clinic or hospital has an AMS program (Table 2).

Participants frequently (43.4%; 129/298) reported being involved in prescribing practices at their facility (technician/nurse 50.8%, student/intern 25.0%, other support staff 38.3%, *p* = 0.001). Most agreed that they are confident in educating clients about AMD use in pets (77.2%; 240/311) (technician/nurse 85.5%, student/intern 55.1%, other support staff 75.5%, *p* < 0.0001) and that they are comfortable collaborating with veterinarians and other staff (61.8%; 186/301). However, only 19.7% (59/300) agreed that they have a role in AMS interventions at their clinic or hospital (technician/nurse 24.1%, student/intern 9.2%, other support staff 16.7%, *p* = 0.03), and only 30.2% (91/301) agreed that veterinarians listen to their input when prescribing AMDs (technician/nurse 35.6%, student/intern 15.4%, other support staff 29.2%, *p* = 0.009) (Table 2). Only 26.2% (78/298) agreed that they have an impact on AMD prescribing during a veterinary appointment.

Specific to AMD prescribing practices within their hospitals, roughly one-third of participants (34.9%; 104/298) reported that advice about whether a pet needs antibiotics is routinely given to pet owners over the telephone at the time a client makes an appointment for their pet. Across all participants, it was estimated that 82.6% of all antibiotic prescriptions recommended by a veterinarian at the time of examination are filled in the clinic or hospital.

## Discussion

The mitigation of AMR in companion animal medicine is a priority for both animal and human health, and a team approach is instrumental for stewardship interventions to be successful. There is a need to educate veterinary support staff about judicious AMD use in companion animal medicine and involve them in clinical AMS programs. Our study found that veterinary support staff are aware of the problem of AMR but are less aware of AMS principles within companion animal medicine. Regarding the AMD prescription process, staff felt comfortable with AMD client education and veterinarian collaboration but often felt they had little impact on the prescribing process nor had a role in AMS interventions.

Several discrepancies regarding veterinary support staff AMR knowledge and perceptions were noted in this study. First, many support staff professionals were concerned with AMR on a global scale but did not perceive it as a problem at their facility. While no previous studies have assessed AMR knowledge and perceptions of veterinary support staff, findings in human medicine have noted a similar discrepancy between AMR concern and the perceived contribution of their facility to the problem (13–15). A second discrepancy found in the current study was that few surveyed veterinary support staff were familiar with AMS principles in veterinary medicine despite recognizing AMR as a problem and receiving education from their employer that focused on the topic of AMU. Similar findings of relatively higher AMR awareness when compared to AMS knowledge have been noted in previous human medicine studies (13, 14, 16). In contrast, a study of Australian veterinary students noted good understanding of both AMR and AMS, but that there were differences between what they were taught in the classroom versus in clinical training (17). It is unknown whether the lack of AMS awareness is due to the absence of veterinary support staff involvement in AMS programs, differences in AMS principles taught formally versus on-the-job training, or the result of few veterinary facilities implementing AMS programs.

Participants identifying as a veterinary technician or nurse agreed that they played a role in AMS interventions more often than they agreed that they were aware of AMS principles in general. This discrepant finding may indicate that veterinary technicians feel they are performing AMS activities as part of their job but do not fully understand the principles behind those tasks. This finding, along with the contradictions noted above, demonstrates a need to educate support staff about the threat of AMR at the clinic level and to increase awareness of veterinary AMS program principles. Previous assessments noted an improvement in knowledge of AMR and AMS among human healthcare professionals after formal training programs (18, 19). Furthermore, there have been calls in human medicine to make AMS education for support staff a priority (20). Based on data gathered in this assessment, a similar approach to educating veterinary support staff about AMR and AMS standards should be pursued to enhance the success of AMS programs.

The results describing involvement in the AMD prescription process demonstrate that support staff are relatively comfortable educating clients about AMU and confident in collaborating with veterinarian colleagues but feel they have little impact on the AMD decision-making process. At the same time, participants indicated that advice about a pet’s need for AMDs is routinely given to clients prior to the pet being examined by a veterinarian. This may impact client expectations about receiving an AMD for their pet even before a veterinarian has had the opportunity to evaluate a pet’s condition and need for a prescription. We noted that most AMD prescriptions are filled in the clinic as opposed to an outside pharmacy, indicating that the AMD prescription process typically takes place in the absence of other external stakeholders (e.g., pharmacists). These results suggest that there are natural opportunities for support staff to influence judicious AMU both before a veterinary consultation and within hospital walls. The inclusion of all professional staff in the AMD prescription process and AMS activities is emphasized in the human medical field, as studies have concluded that nurses possess the necessary skills to be part of team-based stewardship solutions (21, 22). Nurses in human medicine are also seen as having a critical liaison role between stakeholders (i.e., physicians and patients) in the AMD prescription decision-making process (23). Medical support staff perform numerous functions that are necessary for prudent AMU, including patient communication, drug administration, and collaborating with physicians (20). Additionally, it has been previously noted that human medical staff perceive that they play an important role in AMS activities (24). Given that veterinary support staff perform several functions that can support clinical AMS and are comfortable educating clients about AMD use in their pets, these professionals should always be considered when developing and implementing AMS programs. This involvement may come in the form of delivering continuing education, enhancing collaboration between staff and veterinarians, and assigning AMS roles that match individual support staff member strengths. Further information is needed, however, on the barriers that prevent the formal inclusion of support staff in the AMD prescription process and AMS programs.

This study had several limitations. First, this was a cross-sectional survey, which provides only a snapshot of current attitudes and perceptions among veterinary support staff professionals. Additionally, participants self-selected into the study and may have been different than the target population, resulting in possible selection bias, which could potentially limit the generalizability of the study’s findings. Based on experience and background, participants may have interpreted survey questions differently than intended, potentially introducing an information bias. Next, as we did not collect information on clinic location to protect participant identity, we were unable to account for possible correlations in survey responses clustered by facility. This limitation has the potential to bias estimates away from true null associations, making them appear more significant than they are in the presence of clustering. Finally, only a small number of participants indicated that they worked in the “other” role (i.e., client care representative, groomer, or hospital manager). While these individuals often have less direct contact in the medical care of animals, they may still have an important role in the AMD prescription process. With a low response from this role, it is difficult to define their knowledge of AMU use in companion animals and their perception of involvement in the AMD prescribing process.

## Conclusion

This study addresses a critical knowledge gap within the companion animal AMD prescription process by surveying veterinary support staff, a population that has not been extensively considered in previous research. Veterinary support staff have variable knowledge of AMR and AMS in companion animal medicine, resulting in several discrepancies that demonstrate the need for further education among these professionals. As participants reported a high level of confidence when educating pet owners and collaborating with veterinarians but perceived a limited impact on the AMD prescription process, clinical AMS plans should explicitly incorporate veterinary support staff and assign stewardship tasks that match individual strengths. By emphasizing AMS principles among veterinary support staff and encouraging them to advocate for judicious AMD use, these professionals can play a significant role in improving AMU in companion animal medicine.

## Data availability statement

The raw data supporting the conclusions of this article will be made available by the authors, without undue reservation.

## Ethics statement

The survey was administered anonymously, and no personal information was collected. The study protocol and survey tool were reviewed by the Colorado Multiple Institution Review Board (COMIRB) and found to be “Not Human Subject Research”. Participants self-selected into the study and acknowledged informed consent prior to beginning the survey.

## Author contributions

LG-S: Conceptualization, Data curation, Formal analysis, Methodology, Writing – original draft, Writing – review & editing. DT: Conceptualization, Data curation, Formal analysis, Methodology, Project administration, Writing – original draft, Writing – review & editing. ESW: Conceptualization, Formal analysis, Funding acquisition, Methodology, Project administration, Supervision, Validation, Writing – review & editing.
