# Supplementary material for: Veterinary support staff knowledge and perceptions of antimicrobial drug use, resistance, and stewardship in the United States
Source: Front Vet Sci. 2024 Aug 7;11:1401290. doi: 10.3389/fvets.2024.1401290 (PMC11337104; doi:10.3389/fvets.2024.1401290)
Supplement: Supplementary file 1 [file Data_Sheet_1.PDF]

Your participation in this survey is completely voluntary. Best efforts will be made to maintain the security and confidentiality of the information collected. Risks exist when information is collected online and can be intercepted, corrupted, lost, destroyed, arrive late or be incomplete. If you choose to share any health-related or other information, including sensitive student or employee data, measures have been put in place to ensure confidentiality and minimize risks. If the information you provide requires a response, your identity will be verified before discussion of any potentially sensitive information, specifically including your health-related information. By continuing with this survey, you understand and accept these risks associated with disclosure of your information.

Consent

This nationwide survey aims to assess opinions of antibiotic use in small animal practice. Responses will be used to develop training tools and sustainable antibiotic use plans. The survey should take approximately 5 minutes and all responses are kept ***anonymous and confidential***. Should you have questions regarding the survey, please contact Dr. Dan Taylor DVM, PhD at daniel.d.taylor@cuanschutz.edu

Your responses are valuable and will make a substantial impact on the overall understanding of antibiotic use in small animal medicine.

Please indicate your willingness to participate in the survey below.

Yes, I consent to participate.

No, I do not consent to participate.

Demographics

Do you work, or have you worked, in a veterinary setting (i.e., clinic/hospital, shelter, rescue, grooming, boarding, training, etc) or are you currently in a veterinary-related training program?

Yes

No

Unsure

What best describes your current, or most recent, role in a veterinary setting?

Veterinary technician

Veterinary nurse

Veterinary technician assistant

Technician or assistant student

Hospital management

Client care (i.e., receptionist, client advocate, etc.)

Grooming

Daycare

Pet boarding

Pet training

Student

Other (please specify)

What best describes your **current** employment setting? (Select all that apply)

General small animal hospital

Mixed animal hospital

Emergency hospital

Speciality hospital

Shelter/rescue

Feline only hospital

Mobile practice

Academic teaching hospital

Unemployed

Retired

Grooming, boarding or daycare facility

Student

Other (please specify)

Did you attend a formal training program for your current position (e.g.- veterinary technician program, veterinary assistant program, etc.)?

Yes

No

Unsure

How many years have you worked with animals in a veterinary setting?

Less than one year

1 to 5 years

6-10 years

11-15 years

More than 15 years

What state do you **primarily** work in (based on where you worked the most hours in the last 12 months)?

Which of the following best describes the location of your place of employment?

Urban

Suburban

Rural

Unsure

Prefer not to answer

AMR & AMS Familiarity and Perceptions Block

Definitions for the purpose of this survey:

Antibiotic drugs = medications used to treat infections caused by bacteria.

Antimicrobial resistance (also antibiotic resistance, "AMR") = the ability of bacteria to resist the effects of an antibiotic to which it was once susceptible.

Antibiotic stewardship program (also antimicrobial stewardship, "AMS")= Protocols, programs or other materials that promote appropriate use of antibiotics.

Please respond to the following statements:

	Strongly disagree	Disagree	Neither agree nor disagree	Agree	Strongly agree	Don't know	Not applicable
I am familiar with antibiotic resistance (i.e. AMR).	○	○	○	○	○	○	○
AMR is a global concern.	○	○	○	○	○	○	○
I am concerned about AMR at my clinic/hospital.	○	○	○	○	○	○	○
I am familiar with antibiotic stewardship programs.	○	○	○	○	○	○	○
I am confident educating clients about antibiotic use in their pets.	○	○	○	○	○	○	○
I am aware of antibiotic stewardship programs implemented at my clinic/hospital.	○	○	○	○	○	○	○

Involvement, Support, and Education Block

Please respond to the following statements:

	Strongly disagree	Disagree	Neither agree nor disagree	Agree	Strongly agree	Don't know	Not applicable
I have a role in antibiotic stewardship interventions at my clinic/hospital.	○	○	○	○	○	○	○
I have an impact on whether an antibiotic is prescribed to an animal during a veterinary visit.	○	○	○	○	○	○	○
Veterinarians listen to my input when prescribing antibiotics.	○	○	○	○	○	○	○
I am comfortable collaborating with veterinarians and other staff regarding antibiotic use.	○	○	○	○	○	○	○

Please respond to the following statements:

	Yes	No	Unsure	Prefer not to answer
I am involved in the antibiotic prescription process at my clinic/hospital.	○	○	○	○
I have received education focused on antibiotic use (i.e. from your employer).	○	○	○	○
My clinic/hospital has an antibiotic stewardship program.	○	○	○	○

	Yes	No	Unsure	Prefer not to answer
Advice about whether a pet needs antibiotics is routinely given to a client at the time of making an appointment at my clinic/hospital.	○	○	○	○

In your best estimation, what percentage of antibiotic prescriptions are filled at an outside pharmacy as opposed to at your hospital?

	0	10	20	30	40	50	60	70	80	90	100
Percentage of antibiotic prescriptions filled at an outside pharmacy											

Antimicrobial Stewardship Knowledge and Perceptions Block

Conclusion/Thank You

Powered by Qualtrics
